# ‘Taking care of your mind’: participatory research to identify priorities and co-design wellbeing resources with the Muslim community following COVID-19

**DOI:** 10.1186/s12889-026-28816-w

**Published:** 2026-08-04

**Authors:** Caitlin H Douglass, Ana L Orozco, Remzi Unal, Sherene Hassan, Durre Shahwar, Cate Grindlay, Samsam Geereh, Vivienne Mah, Margaret Hellard, Amy Kirwan, Alisa Pedrana

**Affiliations:** 1 Disease Elimination Program, Burnet Institute, 85 Commercial Road, Melbourne, VIC 3004 Australia; 2 Monash School of Public Health and Preventive Medicine, Monash University, 509 St Kilda Rd, Melbourne, VIC 3004 Australia; 3 Melbourne School of Population and Global Health, University of Melbourne, 207 Bouverie St, Carlton, VIC 3053 Australia; 4 Australian Multicultural Foundation, Level 1/185 Faraday St, Carlton, VIC 3053 Australia; 5 Islamic Museum of Australia, 15a Anderson Road, Thornbury, VIC 3071 Australia; 6 Your Community Health, 125 Blake Street, East Reservoir, Melbourne, VIC 3073 Australia; 7 Department of Infectious Diseases, Alfred Hospital, 55 Commercial Road, Melbourne, VIC 3004 Australia

**Keywords:** Participatory, Multicultural, Public health, Mental health, COVID-19

## Abstract

**Background:**

Throughout COVID-19, public health responses lacked engagement with multicultural communities. Collaborating with multicultural communities is essential to understand priorities and create culturally appropriate solutions. Our participatory study aimed to identify the public health priorities of the Muslim community, explore potential solutions, develop resources and monitor their uptake.

**Methods:**

In 2022, we formed a research-community partnership with the Burnet Institute, Islamic Museum of Australia, Australian Multicultural Foundation and Your Community Health. Together, we co-developed a cross-sectional survey to identify the priorities of a Muslim community in Melbourne, Australia following the COVID-19 pandemic. Participants (*n* = 57) were recruited through the networks of three bilingual project officers and Preston Mosque. Participants reported what they needed to feel safe, supported and healthy, ranking 15 items. Through open-ended responses, participants could elaborate on their support needs. We then conducted two participatory and interactive workshops with eight Muslim community members to explore potential solutions to identified needs. Data were synthesised in Miro and validated through partner meetings. Together, we drew on results to develop a series of resources. We monitored resource uptake through partner interviews (*n* = 4), a document review and descriptive statistics.

**Results:**

Survey results indicated that participants most needed jobs (60%), mental health support (43%) and education (40%) to feel safe, supported and healthy after the pandemic. In open-text responses, participants identified they wanted improved access to psychological wellbeing support for themselves, their families and communities. Workshop participants recommended encouraging mental health conversations, providing support through mosques and raising awareness about mental health support online. Subsequently, we co-designed a facilitator guide to support Muslim community members to lead conversations about mental health. We also developed five videos to promote mental health awareness among the Muslim community. We identified that partners used the facilitator guide in three community workshops, referred 20 people to Mental Health First Aid Training and began offering counselling from Preston Mosque. Videos received > 17,000 YouTube views.

**Conclusion:**

Our findings identified the importance of accessible mental health support among the Muslim community following COVID-19. Our partnership enabled the co-design and sharing of culturally safe mental health resources through preferred community channels.

**Trial registration:**

Not applicable.

## Background

The COVID-19 pandemic exacerbated existing health and social inequalities worldwide [1]. People from multicultural backgrounds faced disproportionately high rates of COVID-19 infections, hospitalisations and mortality [2–6]. Between March 2020 and November 2023, 20,755 people died with or from COVID-19 in Australia [7]. The age-standardised death rate from COVID-19 was higher in people born overseas than those born in Australia (16.3 versus 11.9 deaths per 100,000) [7]. Moreover, people born in the Middle East experienced the highest age-standardised death rate from COVID-19 (31.9 deaths per 100,000) [7], and living in ethnically diverse council areas in Melbourne and Sydney was associated with higher rates of COVID-19 infection and excess deaths [8].

In 2023, the Australian Government COVID-19 Response Inquiry explored ways to improve future pandemic preparedness [9]. The Inquiry reported that people from multicultural communities should be better included in future pandemic planning due to inequitable health and social outcomes [9]. Intersectional factors including employment, socio-economic status, living conditions, and access to health care shaped COVID-19 outcomes [10]. Evidence suggests that multicultural community members were at higher risk of COVID-19 exposure due to overcrowded living conditions, working in essential public-facing roles and insecure employment [6, 9]. They were also less likely to receive early COVID-19 vaccinations [9]; major barriers included insufficient information available in languages other than English and mistrust of governments and health systems [11]. Despite these disparities in pandemic-related health outcomes [11], efforts to improve health and wellbeing among Australian multicultural communities were inadequate [12].

Like many countries, Australia introduced public health measures to reduce COVID-19 transmission [13]. However, partly due to the need to respond quickly, planning was often reactive and neglected engagement with multicultural communities [9, 12]. Studies suggest that pandemic restrictions disproportionately harmed multicultural communities [9, 14]. For example, in Victoria, Australia, the rapid lockdown of public housing residential towers created fear, confusion and stress, particularly among migrants and refugees [13–15]. Throughout the pandemic, multicultural community leaders and service providers identified problems with public health communication [16, 17]. Official resources were often unavailable in community languages or shared through trusted community sources [9, 16, 17]. Translated information was often delayed, contained nonsensical messages or was shared in written format only [16]. Additionally, translations were not always validated with community leaders to ensure content was culturally safe and appropriate, limiting people’s access to public health advice. Multicultural communities also faced exclusion from financial support, with some international students, temporary migrants and asylum seekers ineligible for income support payments [18]. Inadequate engagement and planning with multicultural communities throughout the pandemic increased distrust of governments and health systems and may have contributed to poorer health outcomes [9, 19].

Some multicultural communities faced increased racism, verbal abuse, harassment and exclusion during the pandemic [20]. In Melbourne, people from Muslim communities received significant negative media attention [21]. Unconfirmed news reports claimed that localised outbreaks originated at Eid celebrations, contributing to negative stereotypes about Muslims [22]. A content analysis of social media posts identified that Muslim communities were frequently framed as responsible for COVID-19 transmission by ‘refusing to follow pandemic-related protocols’ [22]. Similar stigmatising discourses also circulated in Western Sydney, with news reports linking COVID-19 transmission to a local mosque [23]. Importantly, these studies urged government agencies to engage with the Muslim community to develop public health emergency solutions that respond to social and cultural norms, experiences and needs [23].

The COVID-19 Response Inquiry recommended strengthening pandemic preparedness by developing partnerships and building trust with multicultural communities [9]. The inquiry recognised that collaborating with multicultural communities was essential to understand their priorities and to create solutions with greater chance of uptake and impact [24]. Given the Muslim community experienced significant harms throughout the pandemic, engagement with the Muslim community is vital to identify priorities and generate contextually specific, acceptable and culturally appropriate solutions for future emergencies [25].

This study aimed to:

1. Understand the local public health priorities of the Muslim community following the COVID-19 pandemic in Melbourne, Australia.
2. Explore ways to achieve the public health priorities of the Muslim community.
3. Co-design outputs with Muslim community partners to achieve public health priorities.
4. Evaluate the uptake of outputs among community partners.

## Methods

### Broader study

This paper describes a study nested within the Victorian Ongoing Initiative for Community Engagement [26]. This program of work used community-based participatory research to determine the public health priorities of multicultural communities following the pandemic in Melbourne, Australia. This paper focuses on our partnership with the Muslim community. 

### Ethics approval

 This study was approved by the Alfred Hospital Ethics Committee (Project 150/22).

### Study design and setting

This study took place in the City of Darebin, a northern suburb of Melbourne [27]. In 2021, Darebin had a population of 148,570 of whom 38% reported using a language other than English at home and 4% identified their religion as Islam [28]. These percentages are similar to wider Victoria (30% and 4% respectively) [28]. People living in Darebin experienced long periods of COVID-19 lockdowns in 2020 and 2021 [29].

We conducted our research in partnership with three local organisations:

- The Islamic Museum of Australia – a not-for-profit foundation that shares education and cross-cultural experiences and showcases Islamic art and architecture [30].
- The Australian Multicultural Foundation, Northern Community Support Group – an organisation that provides support and opportunities for the Muslim community in Melbourne’s northern suburbs to achieve their full potential and a sense of belonging in Australia [31].
- Your Community Health – a not-for-profit organisation that offers medical, dental, allied health, harm reduction and social support services [32].

### Research team roles and reflexivity statement

Our research team brought together community-based and external research perspectives. AK, AO, AP, CD, MH, and VM were researchers with external perspectives, however AO and VM were also young people from multicultural backgrounds who could relate to some experiences of younger participants throughout COVID-19. Strengths of the researchers included experience collaborating with multicultural communities, expertise in design methods, data synthesis skills and generation of practical outputs. Roles included obtaining ethics approval, project oversight, developing the research methods, facilitating workshops, generating data insights, and developing study outputs. CG brought external expertise in health care and working with priority populations. CG supported the development of research methods, study findings and outputs. SG, DS, RU and SH were part of the Muslim community; their strengths included strong community connections, local knowledge, cultural understandings of health and wellbeing and pathways to implement findings. Roles included participant engagement and recruitment, supporting the development of research methods, facilitating and participating in workshops, data interpretation and developing and sharing community outputs. Our combined experiences and strengths enabled us to use culturally appropriate research methods that responded to community priorities.

### Community-based participatory research

We used community-based participatory research to collaborate, generate solutions, create outputs and improve health and social outcomes [33–35]. The ‘Assess Connect Transform In Our Neighbourhood’ (ACTION) framework guided our work [36]. This framework outlines four domains in which communities and researchers work in partnership. ‘Assess’ involves community partners and researchers collaborating to shape the research. We held regular planning meetings to share ideas, knowledge and expertise, and make joint decisions about research direction. ‘Connect’ refers to developing relationships for study implementation and community engagement. Community partners allocated Muslim bicultural workers and other organisational staff to implement the research. Burnet researchers employed a Muslim community engagement officer, whom we supported and trained in culturally appropriate research methods. ‘Transform’ involves sharing results and translating learnings into action. Our research aimed to generate new knowledge and practical, place-based resources that could benefit community members. Partners shared results and next steps with participants in a plain language summary using brief text and images. ‘In our neighbourhood’ refers to adapting the research based on peoples’ experiences and needs. Our research was iterative, building in time and flexibility to reflect on findings and adjust research activities. Given the iterative nature of our work, we divided our study into four phases (Table 1).

**Table 1:** Summary of research phases

Phase	Participants
1. Survey	57 Muslim community members
2. Participatory workshops	8 Muslim health professionals, community partner representatives, community leaders, young people and parents
3. Co-design of resources	Community partners and researchers
4. Evaluation of resource uptake	4 interviews with community partners and a document review

#### Phase 1 (February– August 2022): cross-sectional survey

During initial meetings, our partners described being uncertain about community priorities as the pandemic response changed from strict public health measures to a recovery phase [13]. Partners shared that their community engagement was losing traction and suggested we collect data on COVID-19 experiences, impacts and priorities to shape future service provision. Based on these insights, we co-developed a survey to gain a formative understanding of local needs. Researchers drafted the survey and partners added, changed and removed questions. The final survey included categorical and open-ended questions. Table 2 shows the main variables presented in this paper.

**Table 2:** Survey questions

Question	Data
How has COVID-19 impacted the following in your daily life? Please select ‘Worse than before’, ‘Same as before’ or ‘Better than before’	Categorical: Mental health, caregiving, housing situation, access to food, child’s wellbeing, income, employment financially, employment experience and satisfaction, education access, access to health services, conflicts at home, worries about your health, access to community centres and services
Tell us some more about the changes you experienced	Open-ended responses
What do you, your family and/or your community need now to feel safe, supported and healthy? Please rate in order of which one you need the most (1 being the greatest need right now)	Categorical: Jobs, education, career advice, social connectedness, food, mental health support, housing, primary health, places to go out, area to exercise outdoors, information, childcare, other material support, other
Please explain with more detail about what you, your family and/or your community needs in this area?	Open-ended responses

Partners suggested offering flexible survey participation through online self-completion or in person with a bilingual project officer to support the cultural, linguistic and literacy needs of participants. The survey was programmed in REDCap, a secure web-based platform for data capture, hosted at the Burnet Institute [37]. Researchers trained three bilingual project officers to recruit participants and ask survey questions in Turkish, Arabic and Urdu. Partners promoted the survey through social media, flyers and word of mouth at Preston Mosque. Participants provided informed consent by reviewing an explanatory statement and ticking a box confirming they wished to proceed with the survey. Participants were offered an AUD25 voucher for their time and contributions. The survey was launched in July 2022 and was open for two weeks. We aimed to recruit 50 participants to generate a formative understanding of local needs. This sample size was considered feasible based on community partner advice and in the context of time and resource constraints.

Quantitative categorical survey data were analysed using descriptive statistics and 95% confidence intervals (CI) in Stata version 18 (StataCorp. 2023. *Stata Statistical Software: Release 18*. College Station, TX: StataCorp LLC). Quotes from open-ended survey responses supported and contextualised quantitative findings. To gather reflections and shape Phase 2, we presented results to partners in an online workshop.

#### Phase 2 (September – October 2022): participatory workshops

Survey findings suggested that participants wanted support to improve psychological wellbeing among Muslim families (detailed findings are presented in the results section). Subsequently, we held two participatory workshops to understand how to improve psychological wellbeing among Muslim young people and parents. Community partners recommended recruiting participants who had mental health and social work backgrounds, parents, and young people. Community partners were also invited to co-facilitate and participate in workshops.

Participants were invited to attend both workshops through community partner and social networks at Preston Mosque, the Centre for Muslim Wellbeing, the Islamic Museum of Australia and the Muslim Health Professionals Network. Potential participants expressed interest via phone or email and provided implied consent by attending and contributing to workshops.

Partners recommended we hold workshops at the Preston Mosque, a venue familiar and comfortable for participants. Burnet researchers facilitated sessions with bicultural workers and community partner representatives to ensure participants felt welcomed and included. Both workshops went for three hours, and participants were reimbursed AUD50 per hour. Table 3 outlines workshop activities. Community partners developed culturally sensitive ideas, scenarios and resources that were integrated into the workshops. Most activities involved small group discussion and scribing of key points by facilitators and co-facilitators.

**Table 3:** Workshop activities

Workshop 1	
**Activity**	**Description**
1. Welcome and introductions	• Background information • Introductions to researchers, partners and participants
2. Icebreaker	• Draw an item, place or activity that increases your sense of wellbeing • Share back with group
3. Definitions of psychological wellbeing	• Review definitions of psychological wellbeing • Create culturally appropriate definitions of psychological wellbeing • Share definitions with the group • List key words • Vote on words you value most
4. Understanding the psychological wellbeing of young people and parents	• Work in pairs to review a question • Question 1: ‘What do young people need to improve psychological wellbeing?’ • Question 2: ‘What do parents need to improve psychological wellbeing?’. • Document what is needed and key challenges • Share with broader group • Vote on biggest priorities
5. Exploring ways to improve psychological wellbeing of young people and parents	• Work in small groups to review scenario • Scenario 1: Muslim young person experiencing a mental health challenge • Scenario 2: Muslim parent experiencing a mental health challenge • Create a journey map that shows how the person would access support
6. Evaluation	• Describe a rose (something you liked), bud (something new you learnt) and thorn (something you would change)

**Workshop 2**	
Welcome and summary	• Review summary of key findings • Ask questions and provide reflections
Exploring mental health language	• Review words associated with mental health • Select which words are safe and unsafe
Exploring culturally appropriate mental health support options	• Review list of different services • Consider pros and cons of support options • Identify ways to improve cultural safety of services
Exploring potential resources	• Two groups; one focused on Muslim young people and one on Muslim parents • Review existing mental health resources • Identify examples that were effective and would resonate with their priority population
Evaluation	• Reflect on experience with the research and hopes for outcomes

After each workshop, researchers and community partners held debriefing meetings, where we discussed our initial reflections. Researchers then developed preliminary insights to summarise what we learnt. These insights were shared back with participants at the beginning of each workshop, enabling participants to clarify meaning and share additional reflections.

Workshop data were synthesised using affinity mapping. This approach can be used to visualise large amounts of data into related groups that provide practical, actionable insights [38, 39]. Affinity mapping was appropriate for our study because it enabled us to visually organise data, collaborate with community partners and develop insights to inform design outputs [40, 41]. We transcribed and digitised all content and notes from workshop activities. These data were entered as sticky notes into Miro (2024), an online collaboration tool that enabled us to visualise all our data in one place. Data were originally sorted chronologically by workshop and activity. Researchers AO, AK, CD, VM and SG collaboratively reviewed data during online meetings. Through group discussion, sticky notes were sorted into clusters of similar ideas. Groups of ideas were labelled with a title that reflected a practical action to inform study outputs. We presented preliminary insights to community partners (including authors CG, DS, RU and SH) in three synthesis meetings. Meetings involved open discussions where partners could validate researcher interpretations as well as ask questions, pose alternative ideas and add further suggestions based on local and cultural knowledge. Insights were then updated to include partner interpretations.

#### Phase 3 (October 2022 – October 2023): co-designing outputs

Based on workshop results, we worked with community partners to co-design resources that responded to participants’ priorities and ideas. This process involved three meetings in which community partners and researchers reflected on the data, discussed ideas and produced draft content. Researchers validated ideas by comparing content to workshop data. Community partners further validated ideas through discussions with Muslim community members. Briefly, this process led to the creation of a facilitator guide and five videos (further described in the results section).

#### Phase 4 (February 2023 – June 2025): evaluating the uptake of outputs

To evaluate the uptake of outputs, we monitored how partners shared and used the resources in practice and reviewed further actions taken. In February 2023, we interviewed community partners (*n* = 4) to understand their initial perceptions of resources. In June 2025, we followed up with community partners to review written documents describing resource uptake or sharing, community feedback and partner actions. We reviewed meeting minutes, partner notes and email correspondence. In June 2025, we also reviewed social media and YouTube analytics (including views, likes, average view time and average view percentage from the date videos were posted in September 2023 until June 2025). For quantitative data (activities delivered, video views, likes, average view time and percentage) we present descriptive statistics (raw numbers and percentages). Qualitative data (perceptions, feedback, actions taken) were reviewed by AK, AO and CD. We provide a descriptive summary of content, supported by quotes and notes.

## Results

### Phase one survey results: identifying community priorities

#### Demographics

Fifty-seven people completed the survey. Most participants were female; born outside of Australia (mostly in Egypt, Lebanon or Pakistan); spoke a language other than English at home (mostly Arabic, Turkish or Urdu); reported having children; and reported having COVID-19 at least once (Table 4).

**Table 4:** Demographic characteristics of survey participants

Variable (*N* = 57)	*n* (%)
Age (median, interquartile range)	39 years (31–50 years)
Gender	
Female	41 (72%)
Male	14 (25%)
Missing	< 5
Country of birth	
Australia	21 (37%)
Other^A^	33 (58%)
Missing	< 5
Do you speak a language other than English at home?	
No	15 (26%)
Yes^B^	40 (70%)
Missing	< 5
Do you have children?	
No	13 (23%)
Yes	37 (65%)
Missing	7 (12%)
Are you currently working?	
No	14 (25%)
Yes	41 (72%)
Missing	< 5
Are you currently studying?	
No	47 (82%)
Yes	9 (16%)
Missing	< 5
Have you previously contracted COVID-19?	
No	17 (30%)
Yes	40 (70%)

Percentages may not add up to 100% due to missing data. Due to small cell sizes, the exact number of missing responses are not shown when <5

A. Other countries of birth included Egypt, Lebanon, Pakistan, Germany, Djibouti, Indonesia, Iran, Kenya, Kuwait, New Zealand and Cyprus

B. Other languages spoken at home included Arabic, Turkish, Urdu, French, Hindi, Malay, Sindhi, Somali and Farsi

#### Impacts of the pandemic

**Fig. 1:**
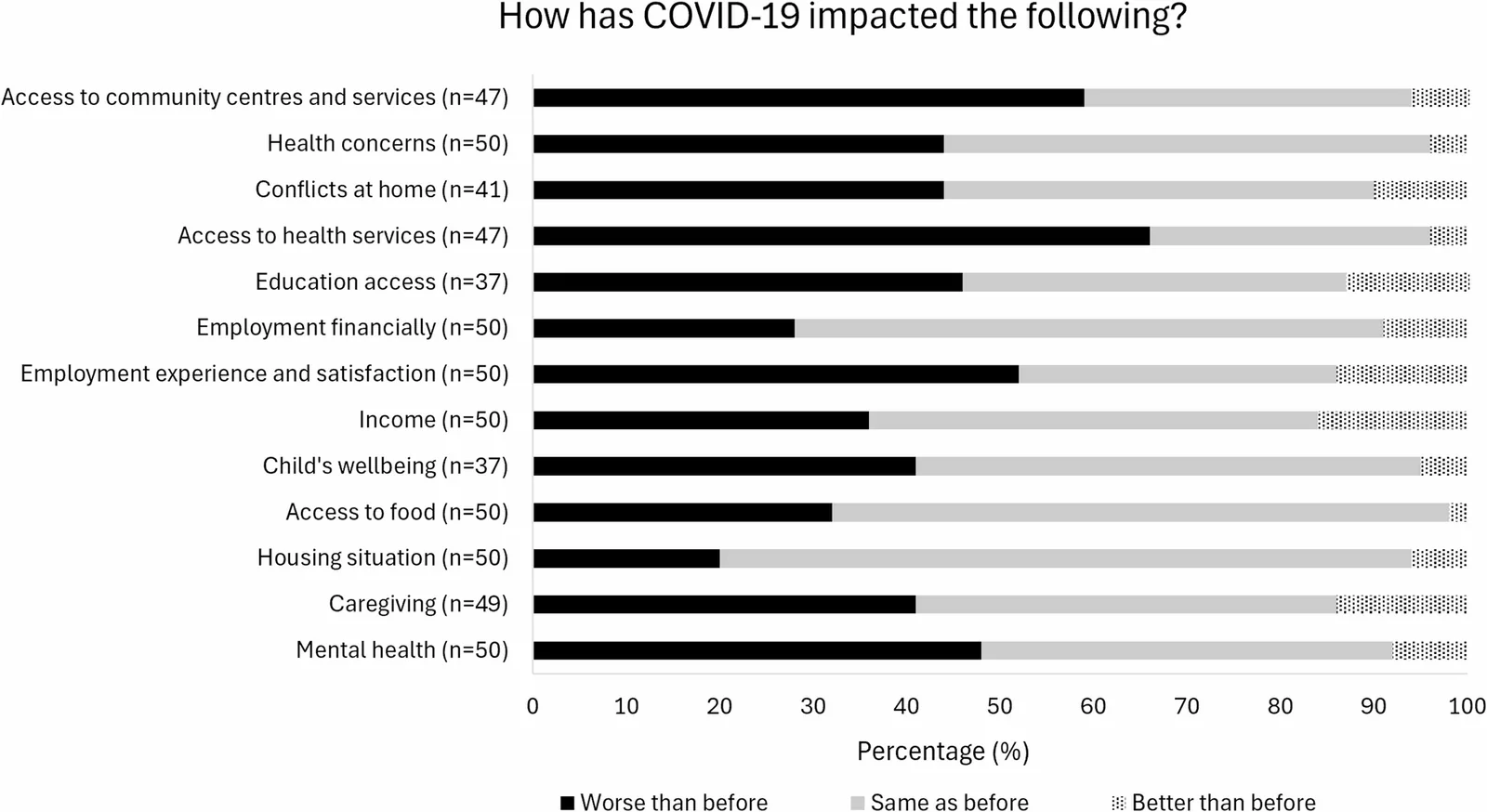
Impacts of COVID-19

Most participants reported that their access to health services (*n* = 31/47, 66%, CI = 52–80%) and community centres (*n* = 27/46, 59%, CI = 44–73%) was worse than before the pandemic. Just under half reported that their mental health (*n* = 24/50, 48%, CI = 34–62%), access to education (*n* = 17/37, 46%, CI = 30–62%), conflict at home (*n* = 18/41, 44%, CI = 29–59%) and health concerns (*n* = 22/50, 44%, CI = 30–58%), were worse than before the pandemic (Fig. 1).

Fifty participants elaborated on pandemic impacts. They described how COVID-19 disrupted their routines, damaging their mental health and social connections. Participants missed significant family and community events, including births, Eid celebrations, and funerals. They felt disconnected from their communities when they were unable to engage in their usual daily activities.

*COVID-19 felt really isolating and restricted*,* which impacted my mental health*,* I couldn’t volunteer or work as much or go to university which felt very isolating as well as having reduced engagement with the community surrounding me*. (Female, 19 years)

Participants described feeling stressed and anxious due to the rising cost of living, lack of affordable housing and insecure employment. Some participants reported they had family members who had lost their jobs or reduced working hours, adding to household stress. Parents reported worrying about their children’s mental health, particularly when they were unable to attend school or work in person.

*Kids’ academic level and wellbeing were terribly impacted. Lockdown was a horrible experience [for them] in terms of mental health. (*Female, 40 years)

#### Support needs

**Fig. 2:**
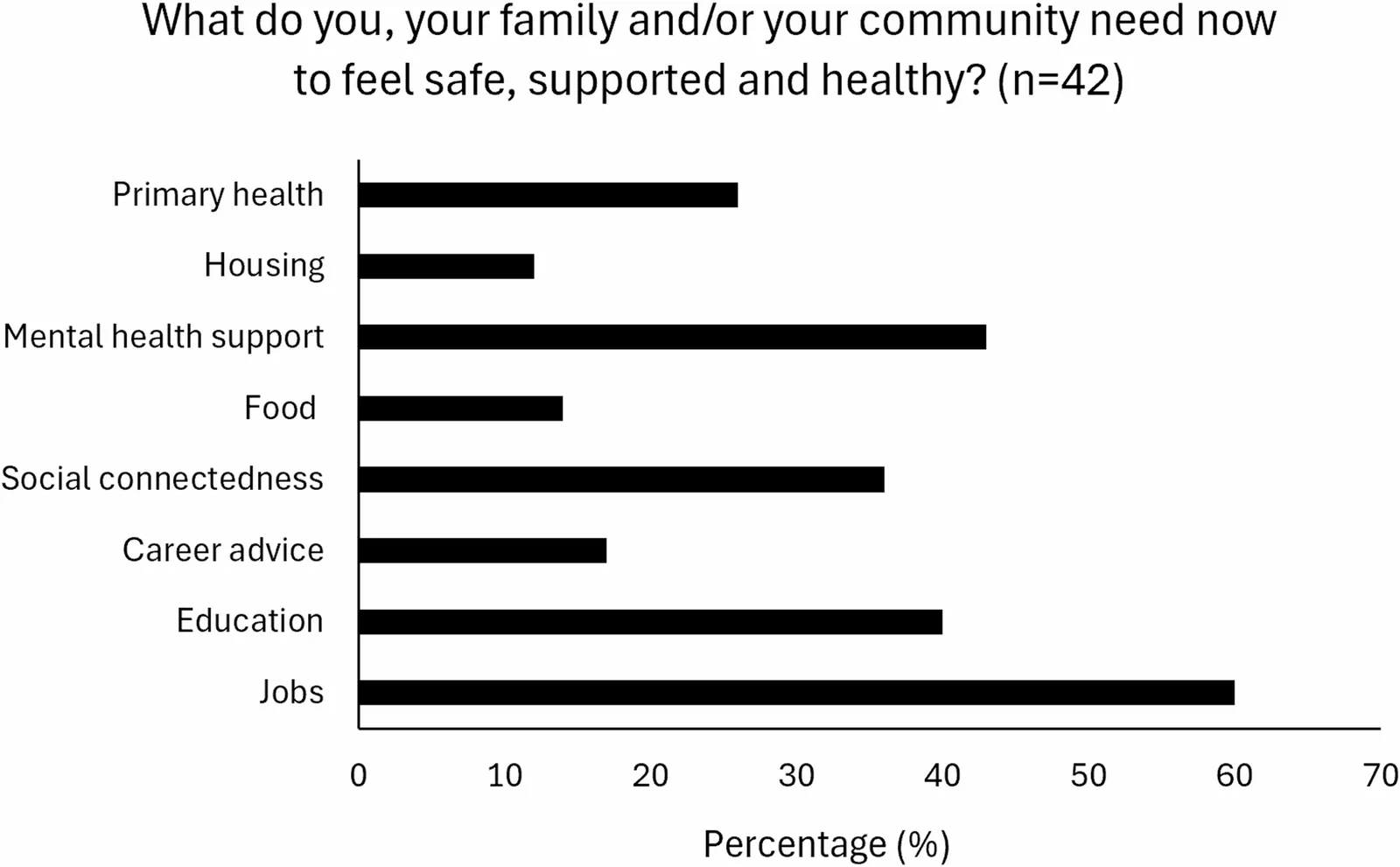
Participants top three needs to feel safe, supported and healthy

Forty-two participants ranked 15 things they needed to feel safe, supported and healthy. Participants’ top three needs were jobs (*n* = 25/42, 60%, CI = 45–74%), mental health support (*n* = 18/42, 43%, CI = 28–58%) and education (*n* = 17/42, 40%, 26–55%) (Fig. 2). Fewer participants selected social connectedness (*n* = 15/42, 36%, CI = 21–50%), primary health (*n* = 11/42, 26%, CI = 13–39%), career advice (*n* = 7/42, 17%, CI = 5–28%), food (*n* = 6, 14%, CI = 4–25%) and housing (*n* = 5/42, 12%, CI = 2–22%) in their top three needs. Fewer than five participants selected training, recreational opportunities, places to go out, information, areas to exercise outdoors, other material support or childcare (data not shown).

Forty-two participants expanded on what they needed to support themselves, their families and communities. Stable employment was central to people’s identity, wellbeing and mental health. During periods of uncertainty and insecurity, participants wanted mental health support so they could look after themselves and their loved ones. They described challenges accessing mental health support for young people during COVID-19, including expensive costs and long waiting times for appointments.

*Easier and quicker access to bulk billed forms of therapy would really assist myself and members of my family and dealing with our own issues in order to reach stronger potential in helping members of this society and one another.* (Female, 22 years)

Other participants shared that culturally appropriate mental health services were urgently needed to reduce stress and anxiety among Muslim families. Participants reported that few mainstream health services offered support in languages other than English. They suggested that information could be shared at mosques to reduce the stigma associated with seeking mental health support.

*There needs to be more information centres*,* maybe at mosques or community centres*,* to give people the opportunity to ask questions*,* get advice from experts about issues / mental health concerns. […] There is a stigma associated with mental health services. Needs to be more culturally sensitive.* (Female, 47 years)

Participants highlighted the need for opportunities to socially connect to protect community wellbeing. As one participant noted, socially connecting was an important mechanism for accessing and providing support to others.

*After being socially isolated during COVID*,* it’s important to build the community again as we rely on each other. Particularly as Muslims*,* we are a small community and we need each other.* (Male, 30 years)

Overall, community partners were surprised that many survey participants raised concerns about mental health and wellbeing, given high levels of stigma in their community. In response, partners suggested we explore ways to improve psychological wellbeing among Muslim young people and parents in phase 2 of the research. 

### Phase 2 workshop results: exploring ways to improve psychological wellbeing

Seven participants attended workshops 1 and 2. One participant attended workshop 2 only (*n* = 8 total). Participants included Muslim health professionals, community partner representatives, community leaders, young people and parents. We generated three practical insights about improving psychological wellbeing among Muslim young people and parents to inform study outputs.

#### Draw on holistic definitions, context and strengths-based language to promote psychological wellbeing

Participants conceptualised psychological wellbeing as holistic, encompassing multiple interrelated domains. Financial wellness was important to feel secure and comfortable to meet life’s demands. Emotional balance equipped individuals with the tools to recognise and cope with emotions. Participants valued spiritual awareness to feel a sense of purpose and connection to Islam. Resilience and *‘knowing your strengths’* were also key parts of psychological wellbeing, allowing individuals to cope with challenges. Participants also believed *‘having a positive self-image’* and *‘feeling connected to community’* were critical for wellbeing. Together, participants drew on these concepts to produce two definitions of psychological wellbeing:

*An individual’s sense of purpose*,* self-acceptance*,* and economic and social security.*

*Overall peacefulness and gratitude*,* with a sense of resilience that mainly comes from spirituality*,* security and connection with family and community.*

To improve psychological wellbeing among the Muslim community, participants suggested that resources should draw on these holistic definitions. Participants recommended avoiding stigmatising labels like *‘mental illness’* and *‘mental disorders’* because they may discourage people from sharing experiences or accessing support. Unfamiliar clinical terms and diagnoses were also perceived as confronting, unsafe and *‘not the right framework’*. Instead, participants recommended adopting strengths-based language that promoted *‘Taking care of your mind’* and *‘Thinking of your body and mind as a gift that you care for’.*

Participants also acknowledged that psychological wellbeing was shaped by contextual factors. Racism and Islamophobia harmed mental health, while connection and belonging protected wellbeing. Participants believed that *‘too much unnecessary pressure set by schools, parents’* and social media had negative impacts on Muslim young people’s self-esteem. For Muslim parents, traumatic migration journeys, limited work opportunities, and financial stress had negative implications on psychological wellbeing.

Participants believed that mental health resources should integrate these broader contextual and cultural factors. For example, resources could include faith-based coping and self-care strategies like prayer, reading the Quran, and connection to Allah. Participants recommended that mental health resources should demonstrate an understanding of cultural experiences and beliefs and use careful and sensitive activities. Including open-ended questions could encourage people to reflect on and share their experiences in their own words.

#### Offer programs and support through culturally safe spaces

Participants reported that seeking mental health support was difficult due to stigma, poor experiences with healthcare services and limited support available in languages other than English. As such, participants identified a need for culturally safe spaces to protect psychological wellbeing and promote support options to Muslim young people and parents. Culturally safe spaces should foster inclusion, kindness and compassion, offer faith-based support options and have diverse, multilingual professionals. The local mosque was an important setting to offer social and wellbeing programs for Muslim young people and parents. For young people, programs could include sports, games, arts-based activities and mentorship. For parents, programs could offer gender-specific and child-friendly peer support groups where parents could discuss family conflict, caring responsibilities and young people’s mental health. Establishing connections with young people and parents in a positive environment could then facilitate conversations and referrals to mental health services.

Participants suggested that Muslim project officers and imams could undergo mental health training to initiate conversations and refer people to further support. One group outlined that their preferred process included *‘Mosque first point of contact. Refer out to trained individuals. Feels safe, comforting’*. Importantly, referral pathways needed to emphasise confidentiality as individuals may experience concerns about privacy and potential social repercussions within family and community contexts. Participants recognised that some community members may prefer to access support through other settings like school, university, their workplace or online to maintain anonymity.

#### Raise awareness of psychological wellbeing and support options

Participants acknowledged that many Muslim community members were unfamiliar with where and how to seek support for psychological wellbeing. Specifically, some parents may have had few opportunities to learn and speak English, making it difficult for them to learn about support services. Participants believed that raising mental health awareness was critical to promote help-seeking and reduce stigma. Participants reported that mental health information should *‘come from a well-known credible source’* like Muslim health professionals or religious leaders, who have the power to speak about taboo topics and resolve misconceptions. Participants were also open to participating in mental health education and training to develop skills to support other community members.

To raise mental health awareness, participants suggested sharing tailored information with the Muslim community through social media. Participants recommended creating simple resources that could be published online and shared through social and community networks. Participants preferred audiovisual material to reach more people and address literacy barriers. YouTube and Instagram were perceived as platforms to reach young Muslims, while Facebook and WhatsApp were seen as effective for reaching Muslim parents. Participants identified they wanted practical resources that outlined how to access culturally appropriate support. Resources could include contact information for support services, answers to common questions and opportunities to learn more. Participants suggested that content should be ‘*free*,* confidential and faith-based*’. Including community stories and personal experiences was also viewed as a powerful way to raise mental health awareness, reduce stigma and promote support services.

### Phase 3 results: co-design of resources

Based on workshop data and community discussions, we co-designed resources that equip people attending Preston Mosque to have conversations about psychological wellbeing. We also developed audiovisual resources to build mental health awareness among the wider Muslim community. Study outputs included a facilitator guide and five videos.

#### Facilitator guide

Researchers drafted a guide for service providers to facilitate conversations about psychological wellbeing with the Muslim community. This draft was shared with community partners to review and provide feedback. The guide includes culturally appropriate images, coping strategies and open-ended questions that enable community members to share what psychological wellbeing means to them. Activities support community members to talk about their experiences and identify their preferred support mechanisms. The guide also acknowledges that community connection is central to psychological wellbeing. Activities are designed to be delivered in group settings to foster social connections and build awareness of culturally appropriate and accessible mental health resources and supports. The facilitator guide is available online [42].

#### Videos

We co-developed a video series titled ‘Let’s Talk About Mental Health’ that addresses open-ended questions including ‘What is mental health?’, ‘How did you support mental health during the pandemic?’ and ‘What advice would you share with young people or parents experiencing mental health challenges?’. The series includes four videos in which credible Muslim community members – a social worker/parent (video 1), an imam/community leader (video 2), a school counsellor (video 3) and a psychologist (video 4) – share clear and practical information about psychological wellbeing. Answers draw on the speakers’ professional and personal experiences and our study findings, including holistic definitions of psychological wellbeing, challenges participants reported, the impact of stigma on help-seeking and the importance of open communication, professional support and self-care. Videos range from 2 to 7 minutes in length and are publicly available on our community partner’s YouTube channel [43].

The Centre for Muslim Wellbeing drew on our findings to develop 'Keeping Safe', a mental health awareness initiative that aimed to encourage help seeking and reduce mental health stigma among the Muslim community [44]. In collaboration with the Sketch Group, they created a short video titled ‘Seeking Help’ [45]. The video uses drawings to show that people from Muslim backgrounds can access support when facing mental health challenges. The video integrates faith-based imagery and references (e.g. ‘Make use of medical treatment, for Allah has not made a disease without appointing a new remedy for it’ and ‘For indeed, with [every] hardship, there is relief’). The video recommends that people visit their general practitioner or the Centre for Muslim Wellbeing to access culturally safe support.

### Phase 4 results: evaluating the uptake of resources

#### Facilitator guide

Our community partners used the facilitator guide to run three group conversations about psychological wellbeing with young women (*n* = 9), female parents of young people (*n* = 16) and male parents of young people (*n* = 12). Sessions were held at Preston Mosque and attendees were reimbursed AUD100 for their time. Our document review identified that the facilitator guide promoted conversations about mental health experiences with diverse community members. Documents suggested attendees preferred activities with visual stimulus and interactions with other community members. Partners reported that some activities were too detailed and time consuming. In future, organisations require resources, training and support to implement the facilitator guide and encourage workshop attendance among community members.

Following the sessions at Preston Mosque, attendees were invited to undertake a Mental Health First Aid training course at the Centre for Muslim Wellbeing. Twenty individuals attended this evidence-based training to develop skills in supporting community members experiencing mental health challenges. Partners felt positive that facilitating discussions encouraged community members to speak openly about mental health.

*The fact that it’s now leading to mental health first aid training […] the people who have done the first aid training*,* how they feel about their own wellbeing and their capacity to help others in their own families*,* in their communities. And that can be a real flow on effect if we’ve got more people […] prepared to have those conversations or feeling that they can.* (Community partner)

Partners believed that facilitating open conversations may contribute to mental health stigma reduction in the Muslim community. However, partners also believed it was important to go beyond discussions and ensure people could get clinical mental health support when needed.

*I just didn’t think there was much therapeutic benefit for those that attended [the facilitated conversations]*,* but having said that*,* it did destigmatise mental health in the Muslim community because it was a platform where Muslims were able to speak about it and you know*,* the more you speak about the topic*,* you know*,* obviously the more we destigmatise.* (Community partner)

In July 2024, partners began offering free in-house counselling and mental health support sessions at Preston Mosque. Sessions are run once per week by a psychotherapist/counsellor, averaging 4–5 bookings. When appropriate, the clinician draws on activities from the facilitator guide to support people to talk about their mental health.

#### Videos

Community partners shared the ‘Let’s Talk About Mental Health’ video series through WhatsApp groups with community members. The Australian Multicultural Foundation also published the videos on YouTube [43]. As of June 2025, the video series had > 17,000 YouTube views (Table 5). The most watched video (video 1) was presented by a social worker advising parents how to support their children’s wellbeing (15,921 views). The ‘Seek Help’ video was also shared on YouTube and social media (Table 4).

**Table 5:** Overview of video analytics

Video	YouTube analytics	Other social media analytics
1 (Let’s talk about mental health - social worker and parent)	Views: 15,921 Likes: 37 Average watch time: 29 s Average percentage viewed: 10%	Shared by the Centre for Muslim Wellbeing on Instagram (522 views, 9 likes) and Facebook (39 views, 4 likes)
2 (Let’s talk about mental health – imam and community leader)	Views: 43 Likes: 0 Average watch time: 1 min 10 s Average percentage viewed: 22%	Shared by the Centre for Muslim Wellbeing on Instagram (604 views, 19 likes) and Facebook (22 views, 3 likes)
3 (Let’s talk about mental health - school counsellor)	Views: 33 Likes: 0 Average watch time: 1 min 37 s Average percentage viewed: 22%	Shared by Centre for Muslim Wellbeing on Instagram (374 views, 9 likes) and Facebook (37 views, 2 likes)
4 (Let’s talk about mental health - psychologist)	Views: 1922 Likes: 9 Average watch time: 27 s Average percentage viewed: 24%	Shared by Centre for Muslim Wellbeing on Instagram (837 views, 29 likes) and Facebook (35 views, 3 likes)
5 (Seek help)	Views: 74 Likes: 0 Average watch time: 1 min 20 s Average percentage viewed: 57%	Shared by the Sketch Group on Instagram (88 views, 3 likes) and Facebook (26 views, 2 likes)

Partners described the videos as relatable and meaningful to the Muslim community, particularly because they used *‘clear and simple language’* and included *‘messaging from trusted leaders’*. During interviews, partners reflected that staff shortages and limited resourcing were barriers to creating additional content and promoting videos with more community members.

*It’s a bit challenging now*,* just trying to find time. We are one staff member short … So that’s my concern. Just getting people to actually open it and creating awareness about it. So I think we need to put a bit of effort into marketing and promoting the material and the resources created.* (Community partner)

## Discussion

This participatory study explored public health priorities and solutions with the Muslim community in the City of Darebin following COVID-19. Our survey identified that participants were concerned about mental health and social connections, particularly for young people and parents. Our participatory workshops identified the need to use holistic definitions of psychological wellbeing, offer support through culturally safe spaces, and raise mental health awareness in the broader Muslim community. We collaborated with community partners to co-design mental health videos and a facilitator guide. Evaluation suggested that videos were shared through preferred community channels. Community partners also used the facilitator guide in community workshops and ongoing practice. Following the research, community partners also referred 20 individuals to Mental Health First Aid training and began offering culturally safe counselling and mental health support from Preston Mosque.

### The importance of a participatory approach

We used the ACTION framework to shape our community-based participatory research [36]. Sharing power and making joint decisions with Muslim community partners was essential because community members had lost trust in government and services during the pandemic [9]. Regular partner meetings, flexibility and listening allowed us to blend researcher and community expertise and develop culturally appropriate research priorities and methods. The strong connections of community partners allowed us to reach community members and explore psychological wellbeing, a highly stigmatised topic within the Muslim community. Adopting an iterative and phased approach allowed us to build trust, check our understanding and adapt the research accordingly. Blending research with community practice helped us to transform knowledge into culturally appropriate and holistic outputs that were shared with community through preferred channels and culturally safe spaces.

### Implications

To embed our findings in the existing literature, we drew on the ‘Muslims Approach to Use of Mental Health Services’, a framework that considers individual and contextual factors that shape mental health support [46]. This framework suggests that demographic factors, cultural beliefs, knowledge, attitudes, stigma, behavioural control and behavioural intentions intersect to shape use of mental health support. Our findings build on the evidence for multiple factors including cultural beliefs, attitudes and perceived stigma and knowledge. 

#### Cultural beliefs

Existing research suggests that Muslim community members prefer accessing support that integrates holistic and spiritual perspectives on mental health [46–49]. Our results reflect these findings, with participants recommending that resources should draw on people’s own definitions of mental health, rather than using a biomedical lens. Our participants emphasised the importance of faith, family and community connections to protect their wellbeing. They suggested that mental health resources should integrate faith-based coping strategies like prayer, reading the Quran and connection to Allah. Other studies have also recognised that migrant, refugee and Muslim communities use faith-based practices to feel comfort, cope with stress and enhance wellbeing [50, 51]. We integrated these cultural definitions into our co-designed resources. Further evaluation is needed to determine the impact of using culturally appropriate language and concepts on mental health help-seeking behaviours.

#### Attitudes toward seeking mental health support and perceived stigma

Existing evidence indicates that mental health issues are stigmatised among the Muslim community, contributing to negative attitudes towards help seeking [46, 52]. Specifically, seeking support has been linked to perceptions of social disgrace among family, friends and community [46]. To challenge these stereotypes and negative attitudes, our participants recommended promoting mental health conversations, offering mental health education and referring young people and parents to mental health services through safe spaces like Preston mosque. Offering support through mosques may challenge stigma by resolving misconceptions, reframing mental health attitudes and encouraging people to access culturally safe care [53]. Other studies suggest that promoting mental health services through trusted Muslim health workers and faith leaders can connect people with training opportunities and professional support [54, 55]. Following the research, community partners ran three mental health workshops, enrolled 20 people into Mental Health First Aid training and began to offer free mental health support and counselling from Preston Mosque. This evidence suggests that promoting mental health support through mosques may be a feasible way to reach the Muslim community and encourage mental health conversations. However, further evaluation of mental health initiatives in faith-based settings is needed to explore the impact on attitudes, stigma reduction and help-seeking behaviours. 

#### Knowledge about mental health support

Existing research indicates that people from Muslim communities may have limited knowledge about mental health and available services [46]. Our participants identified a need to raise awareness about psychological wellbeing and available support for the Muslim community following the pandemic. Participants preferred simple audiovisual resources that were easily accessible by Muslim young people and parents, and recommended sharing mental health information through social media and WhatsApp. Our ‘Let’s Talk About Mental Health’ video series had > 17,000 views to June 2025 – many more than other videos shared by the Australian Multicultural Foundation (49 videos, average 215 views) [43]. These results reflect other research suggesting mental health information can reach people through social media [56]. However, our evaluation found that the average percentage of time viewed for our videos ranged from 10% to 57%. Another study of educational videos found that the average percentage of time viewed was 20–40% [57]. Our data suggest that most viewers did not watch entire videos so engagement levels are unclear. Previous research suggests that mental health videos have higher engagement when they include visuals and personal experiences [56]. Video 5 had the highest average percentage of time viewed; this may be due to its use of animations and Arabic script, while other videos involved individuals speaking to camera. Video 1 had the most views; this may be because the video title described the speaker as a parent, which may have encouraged other parents to click through to learn about their experiences. Further research is needed to determine whether videos can increase mental health awareness among the Muslim community.

### Strengths and limitations

This study was strengthened by our participatory approach. We continuously collaborated with Muslim community partners, enabling us to develop research activities and outputs that reflected community priorities. However, some limitations exist. Our survey had a small sample size and quantitative results lacked precision. Our participants were not representative of all people from Muslim backgrounds in Melbourne, Australia or other broader areas. Results are limited by selection bias as most survey participants were well educated, identified as female and had a median age of 38 years. Additionally, most participants from across the project were connected with Preston Mosque and other community organisations. We likely reached individuals who were interested in wellbeing, community participation and mental health. Participant responses and experiences may not reflect the needs and priorities of younger Muslim people, isolated community members who are not connected with community and faith-based organisations, secular Muslims and recently arrived migrants. As such, our findings should not be generalised to wider populations. Despite this, our research provided a formative understanding of local priorities, with findings strengthened by insights from community partners.

Due to resource constraints, our study documents, workshops and resources were available in English only, limiting the participation of people with other preferred languages. We minimised this limitation by collecting data in partnership with bicultural workers who spoke the same languages as participants. Furthermore, resource constraints meant our evaluation focused on resource uptake including workshop attendance, referrals to mental health first aid training, partner perceptions and video analytics. Although video analytics such as views, likes and watch time provide some evidence of reach and engagement, there are no consistent definitions of success [58]. Further research with more robust measures of effectiveness and impact are needed to understand whether co-designed resources impact mental health literacy, wellbeing outcomes, help-seeking behaviours or stigma reduction. Partners identified that further sharing and evaluation of resources over the long term would require additional funding and support. Research projects that produce practical resources in collaboration with community require long-term funding to support sustainable implementation and evaluation [59].

## Conclusion

We partnered with Muslim community and health organisations to determine the public health priorities of the Darebin Muslim community after the COVID-19 pandemic. We identified that Muslim young people and parents experienced harms to their mental wellbeing and social connections throughout COVID-19. Participatory workshop findings emphasised the importance of improving psychological wellbeing by using holistic definitions, offering services through culturally safe spaces and raising mental health awareness. Working with community partners, we co-designed five videos to raise awareness about psychological wellbeing and a facilitator guide to encourage conversations about mental health. Resources were shared with the Muslim community through preferred channels including Preston Mosque, WhatsApp and social media. Results suggest that offering mental health information and support through mosques may be a feasible way to reach the Muslim community. Our findings provide further evidence of the value of participatory methods when working with multicultural communities. Future community-based participatory research requires additional resourcing to maintain partnerships and evaluate the effectiveness and longer-term impacts of co-designed resources.

## Data Availability

Due to the sensitive nature of the data, supporting data are not available. Resources developed from this study are publicly available online.
